# Human Brain/Cloud Interface

**DOI:** 10.3389/fnins.2019.00112

**Published:** 2019-03-29

**Authors:** Nuno R. B. Martins, Amara Angelica, Krishnan Chakravarthy, Yuriy Svidinenko, Frank J. Boehm, Ioan Opris, Mikhail A. Lebedev, Melanie Swan, Steven A. Garan, Jeffrey V. Rosenfeld, Tad Hogg, Robert A. Freitas

**Affiliations:** ^1^Lawrence Berkeley National Laboratory, Berkeley, CA, United States; ^2^Center for Research and Education on Aging (CREA), University of California, Berkeley and LBNL, Berkeley, CA, United States; ^3^Kurzweil Technologies, Newton, MA, United States; ^4^UC San Diego Health Science, San Diego, CA, United States; ^5^VA San Diego Healthcare System, San Diego, CA, United States; ^6^Nanobot Medical Animation Studio, San Diego, CA, United States; ^7^NanoApps Medical, Inc., Vancouver, BC, Canada; ^8^Miami Project to Cure Paralysis, University of Miami, Miami, FL, United States; ^9^Department of Biomedical Engineering, University of Miami, Coral Gables, FL, United States; ^10^Center for Neuroengineering, Duke University, Durham, NC, United States; ^11^Center for Bioelectric Interfaces of the Institute for Cognitive Neuroscience of the National Research University Higher School of Economics, Moscow, Russia; ^12^Department of Information and Internet Technologies of Digital Health Institute, I.M. Sechenov First Moscow State Medical University, Moscow, Russia; ^13^Department of Philosophy, Purdue University, West Lafayette, IN, United States; ^14^Monash Institute of Medical Engineering, Monash University, Clayton, VIC, Australia; ^15^Department of Neurosurgery, Alfred Hospital, Melbourne, VIC, Australia; ^16^Department of Surgery, Monash University, Clayton, VIC, Australia; ^17^Department of Surgery, F. Edward Hébert School of Medicine, Uniformed Services University of the Health Sciences, Bethesda, MD, United States; ^18^Institute for Molecular Manufacturing, Palo Alto, CA, United States

**Keywords:** brain/cloud interface, brain-computer interface, brain-to-brain interface, brain-machine interface, transparent shadowing, neuralnanorobots, neuralnanorobotics, nanomedicine

## Abstract

The Internet comprises a decentralized global system that serves humanity’s collective effort to generate, process, and store data, most of which is handled by the rapidly expanding cloud. A stable, secure, real-time system may allow for interfacing the cloud with the human brain. One promising strategy for enabling such a system, denoted here as a “human brain/cloud interface” (“B/CI”), would be based on technologies referred to here as “neuralnanorobotics.” Future neuralnanorobotics technologies are anticipated to facilitate accurate diagnoses and eventual cures for the ∼400 conditions that affect the human brain. Neuralnanorobotics may also enable a B/CI with controlled connectivity between neural activity and external data storage and processing, via the direct monitoring of the brain’s ∼86 × 10^9^ neurons and ∼2 × 10^14^ synapses. Subsequent to navigating the human vasculature, three species of neuralnanorobots (endoneurobots, gliabots, and synaptobots) could traverse the blood–brain barrier (BBB), enter the brain parenchyma, ingress into individual human brain cells, and autoposition themselves at the axon initial segments of neurons (endoneurobots), within glial cells (gliabots), and in intimate proximity to synapses (synaptobots). They would then wirelessly transmit up to ∼6 × 10^16^ bits per second of synaptically processed and encoded human–brain electrical information via auxiliary nanorobotic fiber optics (30 cm^3^) with the capacity to handle up to 10^18^ bits/sec and provide rapid data transfer to a cloud based supercomputer for real-time brain-state monitoring and data extraction. A neuralnanorobotically enabled human B/CI might serve as a personalized conduit, allowing persons to obtain direct, instantaneous access to virtually any facet of cumulative human knowledge. Other anticipated applications include myriad opportunities to improve education, intelligence, entertainment, traveling, and other interactive experiences. A specialized application might be the capacity to engage in fully immersive experiential/sensory experiences, including what is referred to here as “transparent shadowing” (TS). Through TS, individuals might experience episodic segments of the lives of other willing participants (locally or remote) to, hopefully, encourage and inspire improved understanding and tolerance among all members of the human family.

## Introduction

“We’ll have nanobots that… connect our neocortex to a synthetic neocortex in the cloud… Our thinking will be a…. biological and non-biological hybrid.”

— Ray Kurzweil, TED 2014

There is an incessant drive in medicine toward the development of smaller, more capable, efficacious, and cost-effective devices and systems. The primary driver of this quest relates to the cellular and sub-cellular genesis of human disease, at which scale, nanodevices can directly interact and potentially positively influence disease outcomes or prevent them altogether, particularly in regard to brain disorders (Kandel et al., 2000, Kandel, 2001; Zigmond et al., 2014; Chaudhury et al., 2015; Fornito et al., 2015; Falk et al., 2016). The pursuit of ever smaller tools to treat patients is approaching a pivotal juncture in medical history as advanced nanomedicine — specifically, medical nanorobotics — is expected to serve as a dynamic tool toward addressing most human brain disorders. The goal is to finally empower medical professionals to treat diseases at individual cellular and sub-cellular resolution (Freitas, 1998, 1999b, 2003, 2005a,c, 2007, 2016; Morris, 2001; Astier et al., 2005; Patel et al., 2006; Park et al., 2007; Popov et al., 2007; Mallouk and Sen, 2009; Martel et al., 2009; Kostarelos, 2010; Mavroides and Ferreira, 2011; Boehm, 2013).

The application of nanorobots to the human brain is denoted here as “neuralnanorobotics.” This technology may allow for the monitoring, recording, and even manipulation of many types of brain-related information at cellular and organellar levels (Martins et al., 2012, 2015, 2016). Medical neuralnanorobots are expected to have the capacity for real-time, non-destructive monitoring of single-neuron and single-synapse neuroelectric activity, local neuropeptide traffic, and other relevant functional data, while also allowing the acquisition of fundamental structural information from neuron surfaces, to enhance the connectome map of a living human brain (Sporns et al., 2005; Lu et al., 2009; Anderson et al., 2011; Kleinfeld et al., 2011; Seung, 2011; Martins et al., 2012, 2015, 2016). Non-destructive neuralnanorobotically mediated whole-brain monitoring coupled with single-cell repair capabilities (Freitas, 2007) is anticipated to provide a powerful medical capability to effectively treat most, or all of the ∼400 known brain disorders, including, most notably: Parkinson’s and Alzheimer’s (Freitas, 2016), addiction, dementia, epilepsy, and spinal cord disorders (NINDS, 2017).

Neuralnanorobots are also expected to empower many non-medical paradigm-shifting applications, including significant human cognitive enhancement, by providing a platform for direct access to supercomputing storage and processing capabilities and interfacing with artificial intelligence systems. Since information-based technologies are consistently improving their price-performance ratios and functional design at an exponential rate, it is likely that once they enter clinical practice or non-medical applications, neuralnanorobotic technologies may work in parallel with powerful artificial intelligence systems, supercomputing, and advanced molecular manufacturing.

Furthermore, autonomous nanomedical devices are expected to be biocompatible, primarily due to their structural materials, which would enable extended residency within the human body (Freitas, 1999a, 2002, 2003). Medical neuralnanorobots might also be fabricated in sufficient therapeutic quantities to treat individual patients, using diamondoid materials, as these materials may provide the greatest strength, resilience, and reliability *in vivo* (Freitas, 2010). An ongoing international “Nanofactory Collaboration” headed by Robert Freitas and Ralph Merkle has the primary objective of constructing the world’s first nanofactory, which will permit the mass manufacture of advanced autonomous diamondoid neuralnanorobots for both medical and non-medical applications (Freitas and Merkle, 2004, 2006; Freitas, 2009, 2010).

It is conceivable that within the next 20–30 years, neuralnanorobotics may be developed to enable a safe, secure, instantaneous, real-time interface between the human brain and biological and non-biological computing systems, empowering brain-to-brain interfaces (BTBI), brain-computer interfaces (BCI), and, in particular, sophisticated brain/cloud interfaces (B/CI). Such human B/CI systems may dramatically alter human/machine communications, carrying the promise of significant human cognitive enhancement (Kurzweil, 2014; Swan, 2016).

Historically, a fundamental breakthrough toward the possibility of a B/CI was the initial measurement and recording of the electrical activity of the brain via EEG in 1924 (Stone and Hughes, 2013). At the time, EEG marked a historical advance in neurologic and psychiatric diagnostic tools, as this technology allowed for the measurement of a variety of cerebral diseases, the quantification of deviations induced by different mental states, and detection of oscillatory alpha waves (8–13 Hz), the so-called “Berger’s wave.” The first EEG measurements required the insertion of silver wires into the scalps of patients, which later evolved to silver foils that were adhered to the head. These rudimentary sensors were initially linked to a Lippmann capillary electrometer. However, significantly improved results were achieved through the use of a Siemens double-coil recording galvanometer, which had an electronic resolution of 0.1 mv (Jung and Berger, 1979).

The first reported scientific instance of the term “brain–computer interface” dates to 1973, ∼50 years following the first EEG recording, when it was envisioned that EEG-reported brain electrical signals might be employed as data carriers in human–computer communications. This suggestion assumed that mental decisions and reactions might be probed by electroencephalographic potential fluctuations measured on the human scalp, and that meaningful EEG phenomena should be viewed as a complex structure of elementary wavelets that reflected individual cortical events (Vidal, 1973).

Currently, invasive^1^ and non-invasive brain–computer interfaces and non-invasive brain-to-brain communication systems have already been experimentally demonstrated and are the subject of serious research worldwide. Once these existing technologies have matured, they might provide treatments for completely paralyzed patients, eventually permitting the restoration of movement in paralyzed limbs through the transmission of brain signals to muscles or external prosthetic devices (Birbaumer, 2006). The first reported direct transmission of information between two human brains without intervention of motor or peripheral sensory systems occurred in 2014, using a brain-to-brain communication technique referred to as “hyperinteraction” (Grau et al., 2014).

The most promising long-term future technology for non-destructive, real-time human–brain–computer interfaces and brain-to-brain communications may be neuralnanorobotics (Martins et al., 2016). Neuralnanorobotics, which is the application of medical nanorobots to the human brain, was first envisaged by Freitas, who proposed the use of nanorobots for direct real-time monitoring of neural traffic from *in vivo* neurons, as well as the translation of messages to neurons (Freitas, 1999b, 2003). Other authors have also envisioned B/CI and predicted that in the future, humans will have access to a synthetic non-biological neocortex, which might permit a direct B/CI. Within the next few decades, neuralnanorobotics may enable a non-destructive, real-time, ultrahigh-resolution interface between the human brain and external computing platforms such as the “cloud.”

The term “cloud” refers to cloud computing, an information technology (IT) paradigm and a model for enabling ubiquitous access to shared pools of configurable resources (such as computer networks, servers, storage, applications, and services), that can be rapidly provisioned with minimal management effort, often over the Internet. For both personal or business applications, the cloud facilitates rapid data access, provides redundancy, and optimizes the global usage of processing and storage resources while enabling access from virtually any location on the planet. However, the primary challenge for worldwide global cloud-based information processing technologies is the speed of access to the system, or latency. For example, the current round-trip latency rate for transatlantic loops between New York and London is ∼90 ms (Verizon, 2014). Since there are now more than 4 billion Internet users worldwide, its economic impact on the global economy is increasingly significant. The economic impact of IoT (Internet of Things) applications alone has been estimated by the McKinsey Global Institute to range from $3.9 to $11.1 trillion per year by 2025. The global economic impact of cloud-based information processing over the next few decades may be at least an order of magnitude higher once cloud services are combined in previously unimagined ways, disrupting entire industries (Miraz et al., 2015). A neuralnanorobotics-mediated human B/CI, potentially available within 20–30 years, will require broadband Internet access with extremely high upload and download speeds, compared to today’s rates.

Humankind has at its core a potent and ceaseless drive to explore and to challenge itself, to improve its collective condition by relentlessly probing and pushing boundaries while constantly attempting to breach those barriers that tenuously separate the possible from the impossible. The notions of human augmentation and cognitive enhancement are borne of these tenets.

This drive includes an incessant quest for exploration and a constant desire for social interaction and communication — both of which are catalysts for rapidly increasing globalization. Consequently, the development of a non-destructive, real-time human B/CI technology may serve as an intimate, personalized conduit through which individuals would have instantaneous access to virtually any facet of cumulative human knowledge and also the optional specialized capacity to engage in myriad real-time fully immersive experiential and sensory worlds.

## The Human Brain

### The Quantitative Human Brain

The human brain comprises a remarkable information storage and processing system that possesses an extraordinary computation-per-volume efficiency, with an average weight of 1400 g and a volume of ∼1350 cm^3^, contained within an “average” intracranial volume of ∼1,700 cm^3^. A brief quantification of the brain’s constituents and operational parameters includes ∼1,350 cm^3^ (∼75%) brain cells, ∼200 cm^3^ (15%) blood, and up to ∼150 cm^3^ (10%) of cerebrospinal fluid (Rengachary and Ellenbogen, 2005). The raw computational power of the human brain has been estimated to range from 10^13^ to 10^16^ operations/sec (Merkle, 1989). The human brain’s functional action potential based information is estimated as 5.52 × 10^16^ bits/sec (Martins et al., 2012), with a brain power output estimated at 15–25 W and a power density of 1.1–1.8 × 10^4^ W/m^3^ at an operating temperature of 37.3°C (Freitas, 1999b).

When considering the human brain at the regional level, an exceptional component is the neocortex (Tables 1, 2), which has a highly organized neural architecture that encompasses sensorimotor, cognitive, and emotional domains (Alexander et al., 1986; Fuster and Bressler, 2012). This cortical structure consists of mini-columnar and laminar arrangements of neurons that are linked via afferent and efferent connections distributed across multiple brain regions (Lorento de Nó, 1938; Mountcastle, 1997; Shepherd and Grillner, 2010; Opris, 2013; Opris et al., 2011, 2013, 2014, 2015). Cortical minicolumns consist of chains of pyramidal neurons that are surrounded by a “curtain of inhibition” formed by interneurons (Szentágothai and Arbib, 1975).

**Table 1:** Neocortical measures (Pakkenberg and Gundersen, 1997; Stark et al., 2007a,b).

	Surface (cm^2^)	Thickness (mm)	Volume (cm^3^)	Neuron number density (10^6^/cm^3^)	Neurons (*N*, 10^9^)
Female	1678–1680	2.61–2.74	440–458	43.1–43.8	19.3–19.7
Male	1883–1900	2.72–2.79	517–524	44.0–44.1	22.8–22.9
Humans	1820	2.69	489	44.0	21.5


**Table 2:** Enumeration of neurons and synapses in the human neocortex (Tang et al., 2001; Sandberg and Bostrom, 2008; Karlsen and Pakkenberg, 2011).

Neocortex region	Total neocortex volume (cm^3^)	Number of synapses (10^12^)	Number of neurons (10^9^)	Number of synapses per neuron (10^3^)	Glial cell number (10^9^)
Occipital	69	22.0	3–4.65	4.36	3
Parietal	149	41.5	4–6.61	6.33	4
Temporal	133	42.0	4–4.80	8.95	5
Frontal	239	58.9	6–7.89	7.54	7
Total	590	164.0	17–23.9	6.93	18


At the cellular level, the average human brain is estimated to contain (86.06 ± 8.2) × 10^9^ neurons, with ∼80.2% (69.03 ± 6.65 × 10^9^ neurons) located in the cerebellum, ∼19% (16.34 ± 2.17 × 10^9^ neurons) located in the cerebral cortex, and only ∼0.8% (0.69 ± 0.12 × 10^9^ neurons) located throughout the rest of the brain (Azevedo et al., 2009). The human cerebellum and cerebral cortex together hold the vast majority (99.2%) of brain neurons (Azevedo et al., 2009). Another approximation, based on combining estimates for the different brain regions, produced a similar value of 94.2 ± 11.3 × 10^9^ neurons for the whole human brain (Martins et al., 2012).

**FIGURE 1:**
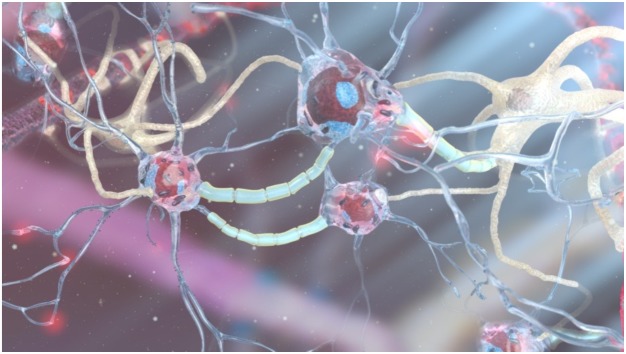
Artistic representation of neurons (with blue processes) and glial (white) cells. [Image credit: Yuriy Svidinenko, Nanobotmodels Company].

Glial cells comprise another brain-cell type (Figure 1). The average number of glial cells in the human brain is estimated to be 84.61 ± 9.83 × 10^9^ (Herculano-Houzel, 2009), with the population of glial cells in the neocortex estimated at from 18.2 to 38.6 × 10^9^ (Karlsen and Pakkenberg, 2011). The ratio of glia to neurons likely has functional relevance (Nedergaard et al., 2003) and varies between different brain regions. While the whole-brain glia/neuron ratio is ∼1:1, there are significant differences between brain domains. For example, the glia/neuron ratio of the cerebral cortex is 3.72:1 (60.84 billion glia; 16.34 billion neurons) but only 0.23:1 (16.04 billion glia; 69.03 billion neurons) in the cerebellum; the basal ganglia, diencephalon, and brainstem have a combined ratio of 11.35:1 (Azevedo et al., 2009).

In addition, synapses, numbering (2.42 ± 0.29) × 10^14^ in the average human brain, are collectively estimated to process information at spiking rates of (4.31 ± 0.86) × 10^15^ spikes/sec, empowering the human brain to process data at (5.52 ± 1.13) × 10^16^ bits/sec (Martins et al., 2012). Synapses are elements of the neural network that play a critical role in processing information in the brain, being involved in learning, long-term and short-term memory storage and deletion, and temporal information processing (Black et al., 1990; Bliss and Collingridge, 1993; Kandel, 2001; Fuhrmann et al., 2002; Lee et al., 2008; Holtmaat and Svoboda, 2009; Liu et al., 2012). Synapses are also key effectors for signal transduction and plasticity in the brain. Proper synapse formation during childhood provides a substrate for cognition, whereas improper formation or functionality leads to neuro-developmental disorders including mental retardation and autism (Rollenhagen and Lübke, 2006; Mcallister, 2007; Rollenhagen et al., 2007). Synapse loss, as occurs in Alzheimer’s patients, is intimately associated with cognitive decline (Dekosky and Scheff, 1990; Terry et al., 1991; Scheff and Price, 2006).

### Processing Units

Structural cellular or sub-cellular elements of the human brain are considered as information processing units if they are involved in significant functional input/output changes in electrochemically based brain-data storage and/or processing systems.

There is some disagreement in the current scientific literature regarding the quantification of this “significance” metric. This incongruity has led various authors to consider different cellular and subcellular structures as fundamental elements of human brain storage and its computation system, encompassing (aside from neurons and synapses): dendritic trees, axons, proteins, and even neural microtubules (Koch et al., 1983; Bialek, 1993; Juusola et al., 1996; Zador, 1998; Manwani and Koch, 2001; London and Häusser, 2005; Ford, 2010).

Estimates for whole-brain electrical data processing rates range from 1.48 × 10^11^ bits/sec. to a high of 3.2 × 10^29^ bits/sec (Sandberg and Bostrom, 2008; Martins et al., 2012). The human brain might even have more than 100 times higher computational capacity than previously thought, based on the discovery that dendrites may generate nearly 10 times as many electrochemical spikes as do neuron soma, and are hybrids that process both analog and digital signals (Moore et al., 2017). This finding may challenge the long-held belief that spikes in the soma (body of the neuron) are the primary means through which perception, learning, and memory formation occur. Dendrites comprise more than 90% of neural tissue, so knowing that they are much more active than the soma would fundamentally alter our understanding of how the brain processes information. As dendrites are ∼100 times larger by volume than neuronal bodies, the immense number of firing dendritic spikes would suggest that the brain may indeed possess significantly higher computational power than earlier estimated.

However, there is currently a consensus that neurons and synapses constitute the fundamental electrochemical processing units of the human brain (Gkoupidenis et al., 2017; Jackman and Regehr, 2017).

The roles of neurons in electrical information processing include receiving, integrating, generating, and transmitting action-potential-based information (Koch, 1997; Koch and Segev, 2000; Zhang, 2008). However, several neuronal noise sources influence the reliability and precision of neuronal signaling, so stimulus-response functions are sometimes unreliable and are dissociated from what is being encoded via spike activity (Bialek and Rieke, 1992).

The other fundamental consensual processing units of electrochemical information are synapses. Synapses are a core component of the neuron network that process information and are involved in learning and memory, with synapse dimensions and morphologies reported as playing a fundamental role in long- and short-term memory storage and deletion. Synapses are also engaged in signal transduction and plasticity, ensuring one-way transmission of signals, and are involved in temporal information processing to allow complex system behaviors, along with acting to decelerate electrical signals (Puro et al., 1977; Black et al., 1990; Bliss and Collingridge, 1993; Kandel, 2001; Rollenhagen and Lübke, 2006; Rollenhagen et al., 2007; IBM, 2008; Lee et al., 2008; Holtmaat and Svoboda, 2009). The role of synapses as processing units of the human brain is reinforced by the results of computational simulation, which indicate that the computational power of a network is increased using dynamic synapses. This suggests that emulation of biological synapses is a prerequisite for the development of brain-like computational systems (Maass and Zador, 1999; Fuhrmann et al., 2002; Kuzum et al., 2012). A recently developed ultra-low-power artificial synapse for neural computing has demonstrated the capacity to provide 500 distinct states (Van de Burgt et al., 2017).

Real-time monitoring of the whole human brain (by placing neuralnanorobots within each neuron and nearby synaptic connections to record/transmit data from localized neuron and synapse spiking) may provide redundant data that might be employed in the development of validation protocols.

## The Cloud

Due to the immense volume of data involved, data transfer to and from living human brains and the cloud may likely require the use of supercomputers with artificial intelligence algorithms. Current von Neumann-based-architecture supercomputers with massive numbers of processors are either centralized (composed of large numbers of dedicated processors) or distributed (based on a large number of discrete computers distributed across a network, such as the Internet).

One estimate of maximum computational speed required to handle the electrical data in the human brain is 5.52 × 10^16^ bits/sec (Martins et al., 2012). Several centralized and distributed supercomputers have processing speeds that are significantly higher than this estimate (Martins et al., 2012). As of November 2018, the fastest supercomputer worldwide was Summit, developed at the United States Oak Ridge National Laboratory (Tennessee), with 122.3 petaflops on the High Performance Linpack (HPL) benchmark. This computational model may be questionable, however, as computers are based on von Neumann architecture, whereas brain circuits are not; and brains operate in a massively parallel manner, whereas computers do not (Nagarajan and Stevens, 2008; Whitworth and Ryu, 2008).

The Internet consists of a decentralized global system, based on von-Neumann-architecture-based computers and supercomputers, used for data transfer across processing and storage units. The global storage capacity of Internet data centers in 2018 was 1450 exabytes (Statistica, 2018). Van den Bosch et al. (2016) estimate that the storage capacity of the World Wide Web doubles every 3 years, with its computational capacity doubling every 1.5 years.

However, once brain data is interfaced with supercomputers in near real-time, the connection to supercomputers in the cloud will be the ultimate bottleneck between the cloud and the human brain (Knapp, 2013). This challenge includes, in particular, the bottleneck of the bandwidth required to transmit data worldwide. According to one study, “Global Internet traffic in 2021 will be equivalent to 127 times the volume of the entire global Internet in 2005. Globally, Internet traffic will reach 30 GB per capita by 2021, up from 10 GB per capita in 2016” (Cisco, 2017). This speed is forcing innovation to deal with bandwidth constraints. Conventional fiber-optic cables transfer trillions of bits/sec between massive data centers. As of October 2018, the average Internet peak connection speed was 189.33 Mbps in Singapore and 100.07 Mbps in the United States (Kemp, 2018). Several commercial efforts to increase Internet speeds are presently underway, including the recently built $300 million fiber-optic cable between Oregon, Japan, and Taiwan. In 2016, much of the world’s Internet traffic was transmitted via undersea fiber-optic cables; the 6,600 km-long MAREA Facebook/Microsoft-owned cable was estimated to carry 160 Tb/sec of data across the Atlantic Ocean (Hecht, 2016). Current commercial 4G networks provide broadband speeds of up to 100 Mbits/sec. However, United States carriers have stated that they plan to deploy 5G technology in 2020 that will eventually “bring speeds of around 10 gigabits per second to your phone. That’s more than 600 times faster than typical 4G speeds of today’s mobile phones, and 10 times faster than Google Fiber’s standard home broadband service” (Finley, 2018).

## Potential of Current Technologies Toward a Brain/Cloud Interface

### Nanoparticles, Nanotubes, and Nanodots

One promising near-term technology that may enable an interface with brain-based neural networks is magnetoelectric nanoparticles, which may be employed to enhance coupling between external magnetic fields and localized electric fields that emanate from neural networks (Yue et al., 2012; Guduru et al., 2015). Magnetoelectric nanoparticles might also induce nanoparticles to traverse the blood–brain barrier (BBB) by applying a direct-current magnetic field gradient to the cranial vault. Magnetoelectric nanoparticles have already been utilized to control intrinsic fields deep within the mouse brain and have permitted the coupling of external magnetic fields to neuronal electric fields. A strategy developed for the delivery of nanoparticles to the perineuronal environment is expected to provide a means to access and eventually stimulate selected populations of neurons (Freitas, 1999b).

The delivery of nanoparticles into the human brain will indeed pose a formidable challenge. For intravenous injection, at least 90% of nanoparticles have been observed to be sequestered within tissues and organs prior to reaching the brain (Calvo et al., 2001), so intra-arterial injections might be more reliable. Steering nanoparticles to selected brain regions may also be achieved using external magnetic fields (Li et al., 2018). Since it has been shown that certain customized nanoparticles may damage dopaminergic and serotoninergic systems, a further detailed analysis of the biodistribution and metabolism of nanoparticles will be required. Further, the risk of infection, inflammatory reactions, potential immunogenicity, cytotoxicity, and tumorigenicity must be effectively addressed prior to the *in vivo* application of nanoparticles in humans (Cupaioli et al., 2014).

The use of carbon-nanotube-based electrical stimulation of targets deep within the brain has been proposed as a novel treatment modality for patients with Parkinson’s disease and other CNS disorders (Srikanth and Kessler, 2012). This strategy utilizes unidirectional electrical stimulation, which is more precise and avoids the surgical risks associated with deep macroelectrode insertion, used with current methods of deep brain stimulation (Mayberg et al., 2005; Taghva et al., 2013) that employ long stereotactically placed quadripolar macroelectrodes through the skull. When intended for use as a component of a B/CI system, carbon-nanotube-based electrical stimulation would also require a two-way information pathway at single-neuron resolution for neuronal electrochemical information recording.

Fluorescing carbon nanodots (synthesized using D-glucose and L-aspartic acid) with uniform diameters of 2.28 ± 0.42 nm have been employed to target and image C6 glioma cells in mouse brains. Excellent biocompatibility, tunable full-color emission, and the capacity to freely penetrate the BBB might make fluorescing carbon nanodots viable candidates as tagging agents to facilitate the implementation of nanomedical B/CI technologies (Zheng et al., 2015). However, fluorescing carbon nanodots might be problematic, since crossing the BBB is a challenging process for ∼98% of all small molecules (Pardridge, 2005; Grabrucker et al., 2016). This is primarily due to the BBB forming a dynamic, blood-and-brain-regulated, strict physical, transport, metabolic, and immunologic barrier while it is permeable to O_2_ and CO_2_ and other gaseous molecules, as well as water and other lipid soluble substances (Serlin et al., 2015), the barrier is very restrictive to large molecules. However, small peptides may cross the BBB by either non-specific fluid-phase endocytosis or receptor-mediated transcytosis (RMT) mechanisms.

Optically based nanotechnologies, including optical imaging methods, have demonstrated valuable applications at the cellular level. For example, quantum dot fullerenes have been employed for *in vitro* and *in vivo* cellular membrane potential measurements (Nag et al., 2017).

### Injectable “Neural Lace”

A recently proposed technology for the potential integration of brain neural networks and computing systems at the microscale is referred to as “neural lace.” This would introduce minimally invasive three-dimensional mesh nanoelectronics, via syringe-injection, into living brain tissue to allow for continuous monitoring and stimulation of individual neurons and neuronal networks. This concept is based on ultraflexible mesh nanoelectronics that permit interfaces with non-planar topographies. Experimental results have been reported using the injection and unfolding of sub-micrometer-thick, centimeter-scale macroporous mesh nanoelectronics through needles with diameters as small as 100 μm, which were injected into cavities with a >90% device yield (Liu et al., 2015). One of the other potential applications of syringe-injectable mesh nanoelectronics is *in vivo* multiplexed neural network recording.

Plug-and-play input/output neural interfacing has also been achieved using platinum electrodes and silicon nanowire field-effect transistors, which exhibited a low interface contact resistance of ∼3 Ω (Schuhmann et al., 2017). Dai et al. (2018) also demonstrated “stable integration of mesh nanoelectronics within brain tissue on at least 1 year scales without evidence of chronic immune response or the glial scarring characteristic of conventional implants.” This group also showed that the activities of individual neurons and localized neural circuits could be monitored and stimulated over timelines of eight months or more, for applications such as recording of alterations in the activities of specific neurons as the brain ages (Dai et al., 2018).

### Neural Dust

Future human B/CI technologies may preferably require long-term, self-implanting *in vivo* neural interface systems, a characteristic that is absent from most current BMI technologies. This means that the system design should balance the size, power, and bandwidth parameters of neural recording systems. A recent proposal capable of bidirectional communication explored the use of low-power CMOS circuitry coupled with ultrasonic delivery of power and backscatter communications to monitor localized groups of neurons (Seo et al., 2013). The goal was to enable scalability in the number of neural recordings from the brain, while providing a path toward a longer-duration BMI. This technology currently employs thousands of independent free-floating 10–100 μm scale sensor nodes referred to as “neural dust.” These nodes detect and report local extracellular electrophysiological data, while using a subcranial interrogator that establishes power and communications links with each of the neural dust elements. Power transmission is accomplished ultrasonically to enable low-efficiency (7%, 11.6 dB) links, yielding ∼500 μW of received power (>10^7^ higher than the ∼40 pW EM transmission available at a similar-size scale) with a 1 mm^2^ interrogator, which may eventually provide ∼10 μm sensing nodes.

### Brain–Machine Interface (BMI)

Brain–machine interface technology is currently being pursued via invasive neural interfaces composed of neural microchip sensor arrays that contain a plurality of electrodes that can detect multicellular signals. These are available for several brain areas (e.g., visual cortex, motor cortex neuroprosthetics, hippocampus, and others) (Berger et al., 2005; BrainGate, 2009).

There are currently two different types of BMI systems. One type samples the neural activity of a single brain and unidirectionally controls an external device (Lebedev, 2014), while the other type (sensory BMI) includes sensory feedback from the device to the brain (O’Doherty et al., 2011). Non-invasive neural BMI interface strategies include the use of EEG, magnetoencephalography (MEG), fMRI (Miyawaki et al., 2008) and optical strategies, including fNIRS (Naseer and Hong, 2015). One 8-channel EEG signal-capture platform, built around Texas Instruments’ ADS1299 analog front-end integrated circuit, may soon be printable at home, thus democratizing low-resolution brain-data-extraction technologies (OpenBCI, 2019).

Neurophotonics integrated with prosthetics, which links artificial limbs and peripheral nerves using two-way fiber-optic communications to enable the ability to feel pressure or temperature, is expected to permit high-speed communications between the brain and artificial limbs. Neuralnanorobots are anticipated to optimize interfaces using advanced touch-sensitive limbs that convey real-time sensory information to amputees, via a direct interface with the brain (Tabot et al., 2013).

At the cellular level, attempts to achieve a direct junction between individual nerve cells and silicon microstructures are being pursued. Neuron-silicon junctions were spontaneously formed using the nerve cells of a mammalian brain, which permitted direct stimulation of nerve cells (Fromherz and Stett, 1995; Offenhausser, 1996; Vassanelli and Fromherz, 1997; Schätzthauer and Fromherz, 1998). Currently, nanoelectronics devices utilizing carbon nanotubes and silicon nanowires can detect and identify neuronal biomolecular chemical secretions and their bioelectrical activities (Veliev, 2016). An array of nanowire transistors can detect, stimulate, or inhibit nerve impulses and their propagation along individual neurites (Freitas, 1999b; Zeck and Fromherz, 2001; Patolsky et al., 2006). To demonstrate experimental minimally invasive neuron cytosolic recording of action potentials, a nanotransistor device was placed at the tip of a bent silicon nanowire to intracellularly record action potentials (Tian et al., 2010; Duan et al., 2011). Vertically arranged gold nanowire arrays have been used to stimulate and detect electrical activity at the nanoscale from simultaneous locations within neurons (Saha et al., 2008). High-density arrays of nanowire FETs enabled mapping signals at the subcellular level – a functionality that is not possible with conventional microfabricated devices (Timko et al., 2010).

In principle, neuralnanorobotics may empower a near-optimal BCI with long-term biocompatibility by incorporating silicon, platinum, iridium, polyesterimide-insulated gold wires, peptide-coated glassy carbon pins, carbon nanotubes, polymer-based electrodes, silicon nitride, silicon dioxide, stainless steel, or nichrome (Niparko et al., 1989a,b; Edell et al., 1992; Yuen and Agnew, 1995; Huber et al., 1998; Malmstrom et al., 1998; Decharms et al., 1999; Normann et al., 1999;Mattson et al., 2000; Kristensen et al., 2001; Parak et al., 2001; Freitas, 2003). Neural electrodes can be implanted without producing any detectable damage beyond the initial trauma and brief phagocytosis, which are typically limited to the edges of the electrode insertion pathway (Babb and Kupfer, 1984) (Freitas, 2003). Several types of neural electrodes are presently employed to interface with the brain via cochlear implants at scala tympani electrode arrays, and in potential CNS auditory prostheses, retinal chip implants, semiconductor-based microphotodiode arrays placed in the subretinal space, visual cortex microelectrode arrays, and other neural implants intended for the mobilization of paraplegics, phrenic pacing, or cardiac assistance (Haggerty and Lusted, 1989; Niparko et al., 1989a,b; Lefurge et al., 1991; Burton et al., 1996; Heiduschka and Thanos, 1998; Guenther et al., 1999; Normann et al., 1999; Peachey and Chow, 1999; Kohler et al., 2001; Mayr et al., 2001; Pardue et al., 2001; Shoham et al., 2001; Freitas, 2003; Mannoor et al., 2013). Each of these electrodes interface with very diminutive and specific brain regions, and are always confined to the surface areas of highly localized domains.

Early “neural dust” proposals for providing BCI access to specific human–brain regions (e.g., neocortex) had several inherent limitations (Seo et al., 2013). Conversely, neuralnanorobotics technologies may possess the appropriate scale for optimally enabling BCI, exhibiting suitable mobility, being minimally invasive, imparting negligible localized tissue damage, and possessing robust monitoring capabilities over distinct information channels without requiring conventional surgical implantation.

Neuralnanorobotics may also be massively distributed, whereas surgically introduced neural implants must be positioned in one or several specific locations. These shortcomings suggest that neuralnanorobotics may be a preferred solution to the formidable challenges ahead in the development of B/CI technologies.

### Brain-To-Brain Interface

**FIGURE 2:**
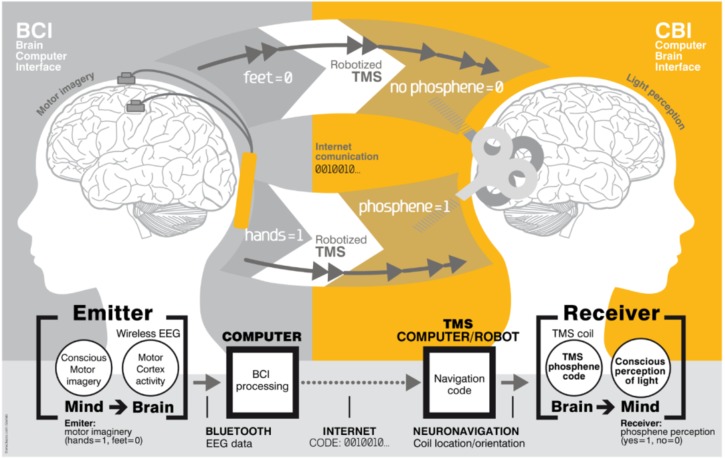
Brain-to-brain interface (BTBI) for information transfer between human subjects. The emitter subject is shown on the left, where sensorimotor cortex activity was recorded using EEG electrodes. The emitter performed an imagery based binary motor task: imagery of the feet (bit value 0) versus imagery of the hands (bit value 1). The receiver subject is shown on the right. The TMS coil was positioned differently over the visual cortex for 1 and 0 bit values, and evoked or did not evoke phosphenes (flashes of light), respectively. An Internet link was used for this brain-to-brain communication. Image reproduced from Grau et al. (2014).

A BTBI involves inducing two distinct brains to directly communicate with each other (Pais-Vieira et al., 2015). BTBI systems were initially implemented in humans (Figure 2) using non-invasive recordings and brain stimulation. Information was transferred from the sensorimotor cortex of one participant (recorded via EEG) to the visual (Grau et al., 2014) or motor (Rao et al., 2014) cortex of the second participant (delivered via transcranial magnetic stimulation, or TMS).

A number of BTBI’s involving different species have also been recently demonstrated, for example, by linking the brain of a human to the spinal cord of an anesthetized rat (Yoo et al., 2013). In another example of interspecies BTBI, a human brain guided the movements of a Madagascar hissing cockroach along an S-shape track, controlling the cockroach antennae via electrical stimulation (Li and Zhang, 2016). Human brains have also been connected to cell cultures, experimentally demonstrating that brain activity can control gene expression, using an EEG-based BMI to trigger optogenetic stimulation of designer cells, thereby mediating their genetic expression (Folcher et al., 2014).

### Brainet Systems

A particularly intriguing application of BTBI technologies, termed “Brainets,” involve the interfacing and processing of neuronal signals recorded from multiple brains, to enable information exchange between interconnected brains (Pais-Vieira et al., 2015) in order to perform cooperative tasks (Ramakrishnan et al., 2015). While not yet particularly sophisticated, recently demonstrated Brainet systems have already provided several interesting insights, including verification of potential direct communications between the brains of two rats located on different continents, after the rats had been permanently implanted with microelectrodes in the sensorimotor cortex (Pais-Vieira et al., 2013).

Experiments have tested three different control systems using 2–3 implanted monkeys that shared BMI-mediated control of a virtual arm (Ramakrishnan et al., 2015). The first type of shared-control, using two subjects, merged recorded neural signals to move a virtual arm on a computer screen. The extracted brain data were summed and observed to improve performance, using noise cancelation. Another system involved two monkeys with partitioned contributions. The first monkey controlled the *X*-coordinate of the virtual arm, whereas the second monkey controlled the *Y*-coordinate. The overall task performance was shown to be improved as each monkey made fewer errors. (Interestingly, each monkey brain adapted and responded less to the other coordinate). A third experiment involved three animals, which together operated and controlled the virtual arm in three dimensions. As the monkeys were unaware that their final task was three-dimensional (given that each monkey had a two-dimensional display) this Brainet might be considered as a rudimentary “super-brain,” where the contributions of individual participants gave rise to higher-order operations that were not performable by each individual alone. Several cooperative BMI schemes have also been implemented in humans — for example, cooperative navigation of a spacecraft (Poli et al., 2013), cooperatively enabled decision making (Eckstein et al., 2012; Yuan et al., 2013; Poli et al., 2014), and movement planning (Wang and Jung, 2011).

A four-brain Brainet system was dubbed an “organic computer” for mimicking simple computer-like operations, such as information-input retention, in a memory-like buffer composed of four serially connected rat brains (Pais-Vieira et al., 2015). This experimental Brainet system always outperformed single-brain computation performance, particularly for discrimination tasks, in which the four brains “voted” to generate the response. This comprised an interesting advance toward the potential eventual emergence of very complex operations in systems with massive numbers of Brainet participants.

A three-human BTBI system, called “BrainNet,” has been recently developed, which allowed three human subjects to collaboratively solve a task using non-invasive, direct brain-to-brain communication (Jiang et al., 2018). Similar to the two-human BTBI system, the three-human BTBI system interface used EEG to record brain signals from the “Senders” and TMS to non-invasively deliver information to the brain of the “Receiver.” The two Senders’ brain signals were decoded using real-time EEG data analysis, extracting their decisions to rotate, or not rotate, a block in a Tetris-like game. These decisions were then uploaded to the cloud and subsequently downloaded and applied to the Receiver’s brain via magnetic stimulation of the occipital cortex. Once this information was received, the Receiver, who could not see the game screen, integrated the information and decided to rotate, or not rotate, the block. The experiment was repeated with five groups with an average accuracy of 0.813. Such high reliability supports further research to improve multi-person BTBI systems that empower future cooperative multi-human problem solving.

Based on current elementary Brainet implementations, it is not yet clear if more complex Brainet systems might be employed for high-throughput information transfer between individual brains, although improved Brainet performance is expected with more advanced Brainet operations. With further progress in the field, the number of information transfer channels may increase, along with the number of subjects involved in each Brainet system. Clinically relevant Brainets that connect patients with therapists, or healthy to unhealthy individuals, would be a particularly interesting application.

### Limited Prospects for Current Techniques

Current technological trajectories appear to be converging toward the creation of systems that will have the capacity to empower a human B/CI. However, since the human brain possesses cellular (neuron) and sub-cellular (synapse) processing elements, any technology that is capable of establishing a long-term and non-destructive, real-time human interface with the cloud must embody the following capabilities: (1) ultrahigh-resolution mobility, (2) autonomous or semi-autonomous activity, (3) non-intrusive (ideally, physiologically imperceptible) ingress/egress into/from the human body, and (4) supplying sufficient and robust information transfer bandwidth for interfacing with external supercomputing systems. Current techniques, whether in present-day or extrapolated future forms, appear to be unscalable and incapable of fulfilling all of the temporal or spatial resolution requirements necessary for a properly comprehensive fully functional human B/CI.

## Neuralnanorobotic Brain/Cloud Interface

Neuralnanorobotics is expected to provide a non-destructive, real-time, secure, long-term, and virtually autonomous *in vivo* system that can realize the first functional human B/CI (Martins et al., 2012, 2015, 2016). Neuralnanorobots could monitor relevant functional and structural connectome data, functional- action-potential-based electrical information processing that occurs within synapses and neurons, and synaptic and neuronal structural changes associated with processing such electrolytic-based functional data (Seung, 2011). Monitoring the intracellular structural and functional connectome may be enabled by three classes of neuralnanorobots, introduced here as endoneurobots, synaptobots, and gliabots (Martins et al., 2016). They also constitute a non-intrusive, self-installed *in vivo* accessory high-speed nanofiber-optic network, which has been described elsewhere (Freitas, 1999b).

More specifically, endoneurobots are autonomous neuron-resident neuralnanorobots that interface with all ∼86 × 10^9^ human–brain neurons at the AIS to directly monitor and interact with action-potential-based electrically processed information. Synaptobots are autonomous neuron-resident neuralnanorobots that might employ multiple flexible stalk-mounted nanosensors to interface with each of the ∼2 × 10^14^ synapses of the human brain to directly monitor and interact with synaptically processed and stored information. Gliabots are glia-resident autonomous neuralnanorobots that are endowed with the capacity to monitor human–brain glial cells and may further serve as supportive infrastructure elements of the system. Subsequent iterations of an initial high-speed nanofiber-optic network may also incorporate wireless transmitters (self-embedded at the periphery of the human brain or within the skull) configured as an evenly distributed network that can wirelessly enable an interface with neurons, axons, and synapses to receive/transmit data from/to the cloud.

To achieve a safe, reliable, high-performance B/CI system, a critical mission requirement is the initial establishment of intimate and stable connections to monitor the electrical firing patterns and waveforms of the ∼86 × 10^9^ neurons and the ∼2 × 10^14^ synapses of the human brain at a suitable repetition rate (400–800 Hz is the reported average maximum range) (Wilson, 1999; Contreras, 2004). Neuralnanorobots themselves, and/or other dedicated nanomedical mapping devices, such as an envisaged Vascular Cartographic Scanning Nanodevice (VCSN) (Domschke and Boehm, 2017) might initially generate an ultra-high-resolution connectome map of the human brain. This would permit the acquisition and storage of detailed structural and functional connectomic data for each unique individual brain and allow for reporting specific spatial coordinates of different classes of neurons, as well as their typical electrophysiological spiking pattern behaviors (i.e., regular-spiking, bursting, or fast-spiking) (Seung, 2011).

For the purposes of a B/CI, interfacing with neuronal and synaptically processed action-potential-based electrical brain activity alone (without monitoring chemically based information) may be sufficient to facilitate robust human B/CI systems. For example, one recent study has found that quantum dots can function as voltage-sensitive probes for real-time visualization of cellular membrane potential in neurons (Nag et al., 2017). Optical interrogation of individual cells and organelles with a spatial resolution of ∼100 nm might be enabled through the use of carbon-nanotube-based endoscopes that project from B/CI nanorobots (Singhal et al., 2011).

Here, synaptically processed action-potential-based information is regarded as fundamental information (Fuhrmann et al., 2002; Shepherd, 2003; Abbott and Regehr, 2004). Synaptobots would detect virtually all of the synaptically processed action potentials and their waveforms and report synaptically processed spikes into the data handling system. Consequently, neuralnanorobots would assist with the prediction of neurotransmitter bursts that traverse each synaptic gap. All these data would be continually processed at sub-millisecond resolution, enabling a virtually real-time data stream between the human brain and the cloud.

### Endoneurobots and Gliabots

**FIGURE 3:**
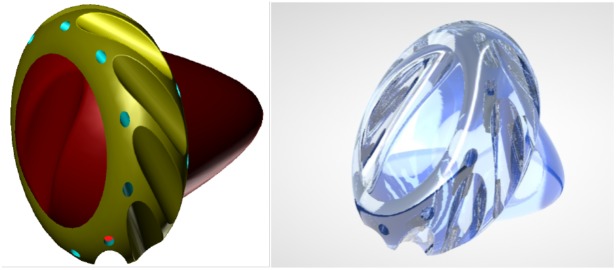
Artistic representation of endoneurobot **(left)** with diamondoid depiction **(right)**. Grooves and orifices might facilitate propulsion within the neurons. Extendable tendrils could project from a number of these orifices to enable stable anchoring and precise post-anchor positioning. [Image credits: **(left)** Frank Boehm - Nanoapps Medical, Inc. and **(right)** Yuriy Svidinenko - Nanobotmodels Company]. (These conceptual illustrations do not literally represent the actual neuralnanorobot design of the endoneurobots).

**FIGURE 4:**
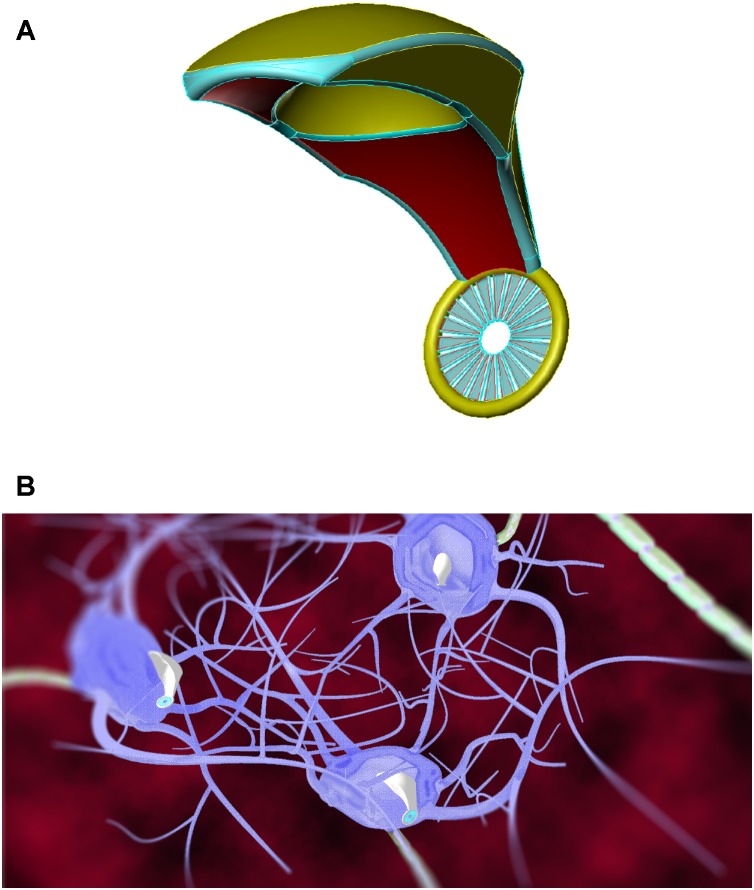
Artistic representations of gliabots, which would self-migrate to glial cells and position themselves intracellularly at the most appropriate intra-glial regions to perform supportive B/CI operations. [Image credits: **(A)** Frank Boehm - Nanoapps Medical, Inc. **(B)** Julia Walker, Department of Chemical Engineering, Monash University]. (These conceptual illustrations do not represent the actual neuralnanorobot design of the gliabots).

Neuralnanorobots might be transdermally injected, after which they would navigate the vasculature and anchor to the endothelial cells of the BBB. A 10 μm^3^ volume of endoneurobots (Figure 3) would subsequently egress the bloodstream, traverse the BBB by methods that have been extensively reviewed elsewhere (Freitas, 2016), enter the brain parenchyma, and begin to navigate within the neuropil. Subsequently, they would enter the neuron cell soma and position themselves intracellularly within the AIS (Martins et al., 2016). Similarly, a 10 μm^3^ volume of gliabots (Figure 4) would egress the bloodstream, enter their respective glial cells, and position themselves intracellularly at the most appropriate intra-glial region, which can vary. The synaptobots would also enter the human body via the bloodstream, cross the BBB (possibly assisted by auxiliary transport nanorobots), enter the brain parenchyma, commence navigation within the neuropil, enter the neuron cell soma, and then proceed intracellularly into the pre-synaptic or post-synaptic structure of a synapse.

The synaptobots would reside in the proper monitoring position within the neurons, in close proximity to presynaptic or postsynaptic structures. Once in place, these neuralnanorobots would monitor the action potentials and the structural changes initiated by the action-potential-based functional data. These data would be transferred from the synaptobots to corresponding endoneurobots (in some cases, with communications and other support from nearby gliabots). Once the data is received by the endoneurobots, it would proceed to the previously installed *in vivo* high-speed nanofiber-optic network, for subsequent transfer to the central units that are responsible for transmitting data to an external supercomputer. The auxiliary nanofiber-optic network system would provide essential support for the data that is transmitted by the endoneurobots and synaptobots, thereby minimizing their onboard data storage capacity requirements. The external supercomputer would communicate with the cloud and handle data post-processing.

An optimal ingress strategy for all species of neuralnanorobots may employ the most rapid route to the human brain through the vasculature. Injection of the neuralnanorobots into the vasculature would be performed in the clinical environment under the supervision of medical personnel^2^. Once injected, the neuralnanorobots would have access to the dense microvasculature of human brain, which is composed of an estimated ∼100 billion capillaries, with a combined surface area of ∼20 m^2^ and a total length of ∼400 miles. Intercapillary distances in the brain are typically ∼40 μm. Hence, each individual neuron within the human brain is at most 2–3 neurons away from a microcapillary (Pardridge, 2011).

The cerebral microvasculature is protected by the BBB, which comprises endothelial cells that are closely abutted as tight junctions. Cumulatively, they form a protective barrier for the human brain that is only naturally crossable by small molecules and lipophilic drugs. Neuralnanorobots can traverse the BBB by methods that have been extensively reviewed elsewhere (Freitas, 2016). For example, the potential uptake of nanoparticles (∼100 nm) through the BBB from the vasculature has been investigated, encompassing numerous strategies including passive diffusion, temporary disruption of tight junctions, receptor mediated endocytosis, transcytosis, and inhibition of *p*-glycoprotein efflux pumps (Kreuter, 2004; Lockman et al., 2004; Agarwal et al., 2009; Hu and Gao, 2010). Since the BBB consists of the endothelium of cerebral capillaries, the choroid plexus epithelium, and the arachnoid membranes (Talegaonkar and Mishra, 2004), it comprises one of the most impermeable ingress pathways for nanomedical devices (100 nm–1 μm) due to the presence of tight junctions.

Once the neuralnanorobots are distributed throughout the brain microvasculature, they could initially seek out any naturally present, randomly placed BBB junctional gaps or imperfections of various dimensions (Freitas, 2003). The BBB is not a perfect barrier, and perijunctional gaps of 0.5 μm have been reported (Stewart et al., 1987; Fraser and Dallas, 1993). Although various strategies exist for the traversal of nanoparticles through the BBB (Freitas, 2003, 2016; Grabrucker et al., 2016), further in-depth study would be required to precisely quantify the population, dimensions, and distribution of naturally occurring perijunctional gaps throughout the BBB network. This would be required if we are to consider passage through the BBB as the most appropriate method of ingress for some B/CI neuralnanorobots.

A process akin to “diapedesis” (the movement of leukocytes out of the circulatory system and toward the site of tissue damage or infection) might be employed by B/CI neuralnanorobots to traverse the BBB. As described by Muller, diapedesis is a multistep procedure by which leukocyte cells cross endothelial cell boundaries from within the bloodstream in ameboid fashion to access sites of inflammation within tissues. In humans, leukocyte transmission through interfacial junctions between tight, laterally apposed (≤0.5 μm thick) endothelial cells involves a number of sequential steps, including the organized activity of molecules upon and within the endothelial cells themselves. Additionally, the dual roles that endothelial cells must play, include facilitating the traversal of (∼7–10 μm in diameter) leukocytes, while sustaining tight apposing seals at the leading and trailing edges of these “passengers” as they are transferred through the junction to negate the leakage of plasma into the interstitial domain (Boehm, 2013; Muller, 2013). Further, it is conceivable that a certain class of facilitative B/CI neuralnanorobots with extendable/telescopic tendrils might project their nanoscopic appendages through smaller nanoscale perijunctional gaps to communicate with those neuralnanorobots that reside on the opposite side of the BBB, within the neocortex itself, or other relevant brain structures (Stewart et al., 1987; Fraser and Dallas, 1993; Freitas, 2003, 2016; Schrlau et al., 2008; Orynbayeva et al., 2012; Boehm, 2013).

Should large BBB junctional gaps be detected by the neuralnanorobots, they may be exploited to penetrate within the neuropil. However, in cases where there is a complete absence of large BBB junctional gaps, mission-designed strategies, including a combination of cytopenetration, cytolocomotion, and histonatation, would likely permit access to the neuropil (Freitas, 1999b, 2003, 2016). The BBB may also be opened using intravenous mannitol (an old method) and ultrasound, externally delivered (Samiotaki et al., 2017; Wang et al., 2017). In addition, “substances may cross the BBB by passive diffusion, carrier mediated transport, receptor mediated transport, and adsorptive transcytosis” (Grabrucker et al., 2016).

Once arrived at their designated neurons, the endoneurobots would autolocate and settle into their monitoring positions, intimately yet unobtrusively. Since action potentials might be initiated in different subcellular compartments, the endoneurobots would be anchored at the AIS (the most likely location for the initiation of action potentials), where they would monitor most action potentials. With some types of neurons, action potentials may be initiated at the first nodes of Ranvier or the axon hillock. Two synaptobots placed at these sites would ensure proper waveform detection of all action potentials. For example, the site of action potential initiation in cortical layer 5 pyramidal neurons is ∼35 μm from the axon hillock (in the AIS). For other classes of neurons, the action potential may be initiated at the first nodes of Ranvier, which for layer 5 pyramidal neurons is ∼90 μm from the axon hillock. The first myelin process is ∼40 μm from soma, whereas the length of the first myelin process is ∼50 μm (Palmer and Stuart, 2006).

All three types of neuralnanorobots (endoneurobots, gliabots, and synaptobots) would monitor action potential-based electrical information using the same types of FET-based nanosensors embedded in their surfaces (Martins et al., 2015). For the monitoring of neuronal structural changes (some of these triggered by the processing of action potentials), once they are securely anchored to the internal neuron membrane surface (with “typical” neurons having a “volume of 14,000 μm^3^ or (∼24 μm)^3^), endoneurobots and synaptobots might employ a tactile scanning probe to image the surrounding membrane surface area of (1.4 μm)^2^ in ∼2 sec at ∼1 nm^2^ resolution (∼1 mm/s tip velocity), or ∼50 s to ∼0.2 nm (i.e., atomic) resolution (∼0.2 mm/s tip velocity), assuming a scan rate of ∼10^6^ pixels/s” (Freitas, 1999b). For their part, gliabots would utilize the same probing strategy.

### Synaptobots

**FIGURE 5:**
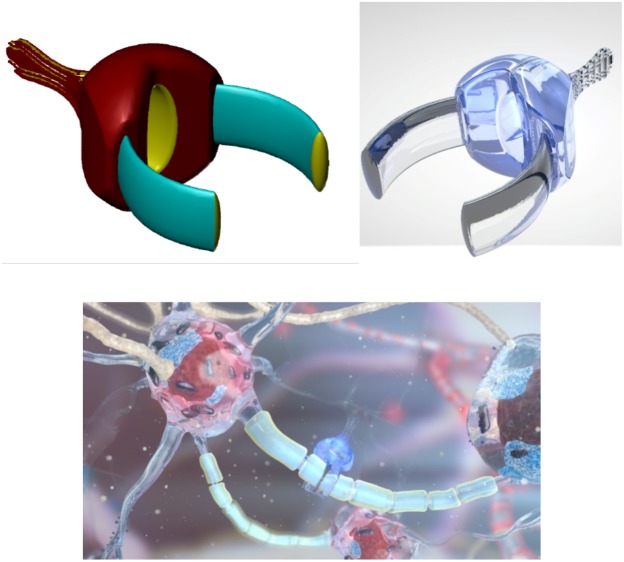
Artistic representations of synaptobot **(left)** with diamondoid depiction **(right)** and calibrating at an axon **(below)**. Oscillating piezo “fins” in conjunction with a central ovoid orifice might enable flow-through propulsion. In one configuration, ultrasensitive extendible/retractable “cuff” nanosensors might externally encircle synaptic gaps to monitor neurotransmitter traffic. [Image credits: **(left)** Frank Boehm, Nanoapps Medical, Inc. and **(right and below)** Yuriy Svidinenko, Nanobotmodels Company. (These conceptual illustrations do not represent the actual neuralnanorobot design of the synaptobots)].

Synaptobots (Figure 5), the most diminutive (0.5 μm^3^) of the three types of neuralnanorobots, are responsible for monitoring synapses, which are relevant sub-cellular structures of the human brain. Synapses (either of the 5–25% electrical or 75–95% chemical variety (DeFelipe and Fariñas, 1992) are key components of the neural network that processes information. They play a crucial role in brain information processing (IBM, 2008) and are involved in learning and memory (Black et al., 1990; Bliss and Collingridge, 1993; Holtmaat and Svoboda, 2009; Liu et al., 2012), long-term and short-term memory storage and deletion (Kandel, 2001; Lee et al., 2008), and temporal information processing (Fuhrmann et al., 2002). They are also the key elements for signal transduction and plasticity in the human brain (Rollenhagen and Lübke, 2006; Rollenhagen et al., 2007). Synapses are so important that proper synapse formation during childhood provides the substrate for cognition, whereas improper formation or malfunction may lead to neurodevelopmental disorders, including various cognitive deficits and autism (Mcallister, 2007). The loss of synapses, as occurs in Alzheimer’s patients, is intimately related to cognitive decline (Dekosky and Scheff, 1990; Terry et al., 1991; Scheff and Price, 2006). The monitoring of synapses is expected to be essential for a stable and robust fully functional real-time B/CI.

Synaptobots would be delivered via the brain microvasculature to avoid long-distance navigation within the brain parenchyma. Auxiliary transport nanorobots having a volume of ∼20 μm^3^ (∼3.2 μm × 2.5 μm × 2.5 μm) might each convey cargos of 24 synaptobots (total of ∼12 μm^3^) through the circulatory system and into the neuron soma. “The full complement of synaptobots would be transported by a fleet of ∼1 trillion auxiliary transport nanorobots, which perform ∼10 round trips to complete the insertion of all synaptobots” toward the implementation of the neuralnanorobotic system prior to the activation of the B/CI system. Individual neurons, on average, would obtain ∼117 such shipments, for an average overall distribution of 2800 synaptobots (≈2.42 × 10^14^ synapses/86 × 10^9^ neurons), which would assign one nanorobot per synapse (Martins et al., 2012).

The protocol for regularly updating the number of synaptobots in the brain (due to nanorobot damage, synapse elimination, neuron death, new synaptic formation, etc.) would be initiated by endoneurobots, which would communicate synaptic requirements to an external supercomputer. About 1 trillion auxiliary transport nanorobots may suffice to accommodate the workload of dynamically adjusting the physical deployment of synaptobots. Auxiliary transport nanorobots (∼2.5 μm) would adhere to a similar transit protocol for crossing the BBB and traversing the neuropil as the endoneurobots and gliabots, which are of comparable size (∼2.2 μm).

Once arrived at the neurons, the auxiliary transport nanorobots would release their cargo of 24 synaptobots into the cytoplasms of each neuron. Following deployment, each synaptobot would either remain within the neuron soma, or navigate (utilizing its onboard locomotion system) from the neuron soma along the axon or dendrite into pre-synaptic or post-synaptic structures — the sites at which synaptic monitoring would occur. To identify and differentiate presynaptic and postsynaptic structures of synapses, synaptobots would initially map (from within the cell) the surfaces of the axon (for axo-axonic, axo-somatic, and axo-dendritic synapses), the neuron soma (for somato-axonic, somato-somatic, or somato-dendritic synapses), and dendrites (for dendro-somatic, dendro-axonic, and dendro-dendritic synapses) (Harris, 1999).

Synaptobots would possess an independent propulsion system for traversing along the axons and dendrites in both directions and may also exploit existing biological neuronal axonic or dendritic transport systems. The process of locomotion may be biomimetically inspired by mitochondrial locomotion strategies within human neurons, to minimize any physiological damage to neuronal processes. Alternatively, oscillating piezo “fins” may operate in conjunction with a ovoid orifice to enable flow-through propulsion for synaptobots (Figure 5). The anticipated synaptobot deployment linear density would be ∼0.5 synaptobots/μm-length of axonic or dendritic processes, and the deployment volumetric number density would be ∼0.5 synaptobots/μm^3^ of axonic or dendritic processes. Maximum synaptobot velocities of ∼1 μm/s may be required to respect biocompatibility requirements, given that the bidirectional movements of mitochondria within axons and dendrites are reported to have velocities of 0.32–0.91 μm/s (Morris and Hollenbeck, 1995; Macaskill et al., 2009), with mitochondrial motility in non-transgenic (NTG) neurons reported as 0.93 ± 0.55 μm/s for anterograde motion and 0.97 ± 0.63 μm/s for retrograde motion (Trushina et al., 2012).

Once securely emplaced at the monitoring positions in close proximity to presynaptic or postsynaptic structures, the primary synaptobot mission would be to monitor the exact timing and intensity of the electrical action potential information arriving at the synapses, and regularly monitor associated changes that occur in key structural elements of the synapse. With one synaptobot positioned near each synapse in the human brain, the action potential data might be acquired using ∼3375 nm^3^ FET-based neuroelectric nanosensors (Martins et al., 2015), enabling monitoring of the synaptically processed 4.31 × 10^15^ spikes/sec. Data collection would have a temporal resolution of at least 0.1 ms, which is sufficient for waveform characterization, even at the maximum human neuronal firing rate of 800 Hz. Facilitated and mediated by endoneurobots and gliabots, the synaptobots would subsequently transmit 5.52 × 10^16^ bits/sec of continuous action potential data (Martins et al., 2012) via an *in vivo* nanofiber-optic network system, as described above (Freitas, 1999b).

Protocols for the application of the B/CI should include regular structural scanning of the human–brain connectome. The synaptobots, along with the endoneurobots and gliabots, could map and monitor relevant neuronal and synaptic structural changes using tactile scanning probe nanosensors (Freitas, 1999b) with special scanning tips that permit the synaptic bouton volume and shape to be measured, along with other relevant synaptic structural characteristics. This structural scanning process may include mapping the main ultrastructural components of a chemical synapse (whether located within the presynaptic axon terminal, the synaptic cleft, or post-synaptic terminal), the postsynaptic density (PSD), the active zone (AZ), synaptic vesicles (e.g., coated vesicles, dense core vesicles, and double-walled vesicles), endoplasmic reticulum, mitochondria, and punctum adhaerens (PA).

While scrutinizing synaptic structural changes, neuralnanorobots would also detect induced changes via monitoring synaptic plasticity and crosstalk, including long-term synaptic based potentiation (LTP), long-term depression (LTD), short-term plasticity, metaplasticity, and homeostatic plasticity. For instance, the activity-dependent modification of PSD proteins occurring over timescales of seconds to hours is believed to underlie plasticity processes such as LTP and LTD (Sheng and Hoogenraad, 2007). Longer-term changes in the PSD structure and composition (from hours to days) involve altered protein synthesis, either within the neuronal cell body, or dendrites (Sheng and Hoogenraad, 2007). The degradation of PSD proteins via the ubiquitin-proteasome system (Bingol and Schuman, 2006) also sculpts the PSD structure and plays a primary role in synaptic plasticity. Remarkably, recent evidence points toward the rapid exchange of PSD proteins, such as AMPARS and PSD-95, even between neighboring synapses under steady-state conditions (Sheng and Hoogenraad, 2007).

Neuralnanorobotic monitoring of the PSD appears to be an essential requirement. The PSD is a complex molecular machine that dynamically alters its structure and composition in response to synaptic activity. The PSD dynamically regulates its components through protein phosphorylation, palmitoylation, local protein translation, the ubiquitin-proteasome system for protein degradation, and redistribution of specific proteins (e.g., CaMKIIα, AMPARs) both entering and leaving the PSD (Kim and Ko, 2006; Sheng and Hoogenraad, 2007). Signaling pathways are organized by PSD proteins to coordinate synaptic structural and functional changes. These proteins also regulate the trafficking and recycling of glutamate receptors (which determine synaptic strength and plasticity), promote the formation and maturation of excitatory synapses by co-aggregating with post-synaptic cell adhesion molecules, organize neurotransmitter receptors within the synaptic cleft, serve as a signaling apparatus. These proteins are also an essential component of an extraordinary synaptic signaling and regulatory assemblage. The “typical” PSD consists of a disk-like structure with an average diameter of 300–400 nm (range 200–800 nm), a thickness of 30–60 nm (Baude et al., 1993; Rácz et al., 2004; Okabe, 2007; Sheng and Hoogenraad, 2007), volume of ∼7.5 × 10^6^ nm^3^, and mass of ∼1.1 GDa (Chen et al., 2005).

Events involving LTP and LTD structural changes to dendritic spines can alter spine number, size, shape, and subcellular composition in both immature and mature spines (Bourne and Harris, 2008). The dendritic spine neck serves as a diffusion barrier (controlled by neuronal activity) to current flow and diffusion of molecules between the spine head and the dendrite. The geometry of the spine neck determines the rate of calcium efflux into the dendrite shaft and hence the degree of elevation of calcium concentrations within the spine head, following *n*-methyl-D-aspartate receptor (NMDAR) activation (Bloodgood and Sabatini, 2005; Alvarez and Sabatini, 2007; Sheng and Hoogenraad, 2007). In experimental work, dendritic spines that received LTP induction increased in volume, from 50 to 200% (Alvarez and Sabatini, 2007), with this increase persisting for more than 1 h following stimulation (Alvarez and Sabatini, 2007). Sustained head enlargement in dendritic spines is induced by LTP, due to F-actin polymerization. LTD causes α-amino-3-hydroxy-5-methyl-4-isoxazole propionic acid (AMPA) receptor internalization with spine elongation and/or shrinkage of spine heads, due to actin depolymerization (Bourne and Harris, 2008).

There exists a clear and strong association between synapse bouton size/shape and the organellar and macromolecular changes that occur within the bouton. This provides some level of information redundancy and suggests that monitoring all dendritic spine organelles and molecular components is likely unnecessary. Synaptobots may deduce a great deal of useful information subsequent to scanning the gross volume and shape of the spine. This information redundancy is expected to significantly reduce synaptobot monitoring tasks.

**FIGURE 6:**
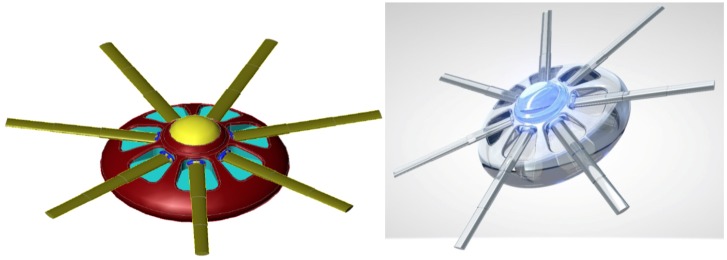
Artistic representations of wireless nanoscale transmitter **(left)**, and in its diamondoid form **(right)**, which might interconnect to form an evenly distributed mesh network, subsequent to self-embedding at the periphery of the brain, on or within the skull. [Image credits: **(left)** Frank Boehm – Nanoapps Medical, Inc.; **(right)** Yuriy Svidinenko – Nanobotmodels Company. (These conceptual illustrations do not represent the actual neuralnanorobot design of the wireless nanoscale transmitter)].

The auxiliary nanofiber-optic system (Figure 6), coupled with endoneurobot and gliabot data transmission support, would likely serve to minimize the onboard data storage requirements for synaptobots. An onboard synaptobot nanocomputer might be manifest as a ∼0.01 μm^3^ CPU device with ∼100 megaflops processing speed. The total internal volume of onboard synaptobot computation might be 0.11 μm^3^ to fulfill redundancy requirements. Such volume allocation is similar to other nanorobot designs with comparable degrees of mission design complexity (Freitas, 2005b).

### Data Transmission Between Neuralnanorobots and the Cloud

Data from the three types of neuralnanorobots would be selected in real time, based on relevance to a specific use (such as auditory or visual content). The data would also be linked to other selected and related network activities, potentially with neurons in the prefrontal cortex and with mixed-selectivity neurons, which have been found to encode distributed information related to task-relevant aspects (Rigotti et al., 2013). Key design goals include: reducing latency, heat buildup, device size, and power for electronics; and tradeoffs for processing and latency between embedded/wearable/portable devices, local processing, and the cloud.

One key future technological advance in reducing latency will be 5G mobile telecommunication, expected in the year 2020 (AT&T Business, 2018). 5G promises to ensure a new way for mobile users to experience VR and AR, for example, via the cloud without latency artifacts. “To give you a sense of scale, the typical refresh speeds for a computer screen are approximately 80 ms” (Weldon, 2016). “However, for AR/VR, the industry is driving the conversation toward the Vestibulo-Ocular Reflex (VOR) — the neurological process by which the brain coordinates eye and head movements to stabilize images on the retina. This is critical to synchronizing virtual and real objects to create a coherent view. The entire VOR process takes the brain 7 ms, a more than 10× reduction over screen-to-brain propagation. … Today’s VR systems recommend a latency of <20 ms for standard performance, and very low latency (<7 ms) is even better. For this reason, developers and inventors want even lower latency to realize what they envision for the next iterations of VR.” Similar performance increases may be found useful in B/CI neuralnanorobotic systems.

### Biocompatibility of B/CI Neuralnanorobotic Systems

Experimental data has provided a wide range of measured human intracranial volumes (1152–1839 cm^3^) and total average cerebral spinal fluid (CSF) volume (82–125.3 cm^3^), with a total brain-cell parenchyma volume of 1319 cm^3^, including 489 cm^3^ of white matter and 786 cm^3^ of gray matter (Vaidyanathana et al., 1997; Nopoulos et al., 2000). During B/CI operations, one ∼10 μm^3^ endoneurobot would reside within every brain resident neuron, giving a total endoneurobot volume of 0.86 cm^3^, or only ∼0.06% of total brain volume. A similar volume will be displaced by gliabots, given one 10 μm^3^ gliabot within each of the 84.6 × 10^9^ brain-resident glial cells (Azevedo et al., 2009), displacing another ∼0.06% of brain volume. Thus, total volume displaced by endoneurobots and gliabots would be ∼0.12% of a “typical” ∼14,000 μm^3^ neuron volume, which is orders of magnitude below the total 1–10% “safe” tissue and organ intrusiveness limit for nanorobots that has been recommended elsewhere (Freitas, 2003).

The synaptobot population represents a more significant neuralnanorobot intrusion on the volume of the human brain. Each synaptobot might contain ten 3375 nm^3^ neuroelectrical nanosensors (Martins et al., 2015) for monitoring the action potentials of up to ten distinct synapses at adequate temporal resolution. Tagging all 2.42 × 10^14^ synapses in the human brain with one robot each would require 24–242 × 10^12^ synaptobots at 0.5 μm^3^ per robot, giving a total fleet volume of 1.2–12 × 10^13^ μm^3^ or 12–120 cm^3^ and representing ∼0.9–9% of total brain volume-just within the “safe” tissue and organ intrusiveness limits.

Neuralnanorobotics for B/CI missions should include the capacity to navigate intracellularly (Martins et al., 2016), and even extracellular navigation might sometimes be required when intracellular navigation is deemed physically difficult, or impossible. For example, certain axonal and dendritic domains are less than 0.50 μm in diameter (Shepherd and Harris, 1998), and myelinated axons at three different sites of the corpus callosum in the human brain are estimated to have axon diameters of ≥0.50 μm, in 70–90% of cases (range 0.16–3.73 μm, mean 0.73 ± 0.55 μm) (Liewald et al., 2014). Thus, a small percentage of the 0.5 μm^3^ synaptobots might encounter difficulty in accessing distal axonic and dendritic regions via strictly intracellular navigation.

The biocompatibility of currently available BCI technologies has been a major challenge. Systems have performed well during acute recordings, but failed to function reliably over clinically relevant timelines, the result of brain tissue reaction against implants, making biocompatibility of implanted BCI systems a primary concern in current device design (Polikov et al., 2005; Winslow and Tresco, 2010; Tresco and Winslow, 2011). Biotic–abiotic interface cell biology has to take into consideration factors pertaining to various scientific domains, including chemistry, cellular biology, physiology, bioelectricity electrochemistry, anatomy, surgery, and microbiology, as well as mechanical factors (Prodanov and Delbeke, 2016). The main reasons for biocompatibility problems with currently available BCI systems derive from induced acute injury, including: the breaching of the BBB to insert devices, the introduction of mechanical tissue strain from volumetric tissue displacement, mechanical tear of cells and the extracellular matrix, the activation of glial cells, the loss of local perfusion, vasogenic edema, secondary metabolic injury, steric blockade of signaling molecules, microglial activation, and locally induced neuronal degeneration (Gunasekera et al., 2015; Jorfi et al., 2015; Kozai et al., 2015). Some strategies have been proposed to address these biocompatibility problems, for example, the manipulation of BCI device surfaces that interface between intracellular and extracellular environments has helped passively reduce local inflammation, and consequently prevent numerous biocompatibility problems (Skousen et al., 2015; Oakes et al., 2018).

With proper design—respecting the limits of volumetric tissue displacement, minimizing residual impact on local perfusion, and ensuring no vasogenic edema—neuralnanorobots are not expected to induce localized acute injury and disruption to the BBB. Neuralnanorobots are also not anticipated to activate microglial immune reactions.

### FDA Protocols for Neuralnanorobotics

The development and implementation of a neuralnanorobotically mediated human B/CI will require that all hardware and software technologies involved in the process are extensively tested, verified, and certified by the appropriate technical and administrative organizations, to ensure compliance with the required protocols for biocompatibility, safety, redundancy, security/privacy, stability, and durability. Selected ingress and egress strategies will also be required to undergo highly detailed and rigorous scrutiny, in alignment with current/downstream FDA approval protocols for proposed clinical nanomedical technologies, particularly those that are to operate within the human brain.

The implementation protocols for neuralnanorobotics may be similar to those currently employed for the approval of any medical technology. The approval mechanism for neuralnanorobotics is expected to include testing the entire system, using (1) computational modeling, (2) laboratory testing, (3) *in vivo* animal studies, (4) robotic avatar testbeds, and (5) human trials. This step-by-step approach will comprise the proper clinical protocols, to be supplemented with detailed risk analysis and mitigation strategies. Once engaged in clinical trials, protection measures for human subjects may be instituted along with proper monitoring, in compliance with the requirements of a data–monitoring committee.

Aside from the FDA approval process, and prior to implementation, all stages of the neuralnanorobotically mediated B/CI system will require that each of its components and systems intended for ingress and egress undergo the comprehensive review of an ethics board. From an environmental perspective, all of the neuralnanorobots are expected to be made of diamondoid materials (likely produced in nanofactories via molecular manufacturing) with all nanodevices being completely recyclable, so they would impart no damage to natural ecosystems or the environment at large. Any disposal quantities should be of negligible volume and chemically inert.

The UN has recently condemned Internet access disruption as a human rights violation (United Nations Human Rights Council, 2016). Similarly, a neuralnanorobotics-based brain cloud interface might also, in the future, be considered a human right, given its profound relationship with the promotion, protection, and enjoyment of human rights on the Internet. The exercise of the human right to freedom of expression on the Internet has been considered of crucial importance, especially during a rapid pace of technological development, supported by the empowerment of individuals from all over the world to use new information and new communication technologies (United Nations Human Rights Council, 2016). In particular, the neuralnanorobotics based B/CI is expected to provide vast opportunities for affordable and inclusive education globally, consequently becoming an important tool to facilitate promotion of the right to education. A comprehensive analysis of the core ethical questions associated with implementation of the neuralnanorobotics-enabled brain cloud interface is expected to precede its implementation and mass adoption.

## Human Brain/Cloud Interface Applications

### Significant Improvement of Education

Cumulative human knowledge doubled approximately every century until 1900. By 1950, human knowledge was doubling every 25 years. As of 2006, on average, human knowledge was doubling every 13 months, and the “Internet of Things” is expected to further lower the doubling time of human knowledge to 12 h (Coles et al., 2006). Such massive amounts of information increase the urgency to radically improve human learning capacities, which are currently limited by biological evolution-driven characteristics. The impracticability of keeping up with the modern rate of creation of scientific knowledge is clearly evident, assuming present-day human biological cognitive abilities (Larsen and von Ins, 2010). Contemporary approaches to this problem include limited strategies such as data mining and research maps (Landreth and Silva, 2013). Neuralnanorobotics may enable us to far surpass our presently limited cognitive capacity to learn in a world driven by exponentially expanding knowledge.

The ultimate learning process may be manifested as direct transfer of knowledge to the human brain, where neuralnanorobots empower practically instantaneous and nearly perfect learning. However, the injection of facts and accumulated knowledge may not necessarily translate to cognition, understanding, meta-analysis or meta thought that can inspire imagination and creativity. Complex skills such as playing the piano or performing a complex brain operation might be “injected” into the brain, which may reduce the time that it traditionally takes to learn the piano, or to be a proficient brain surgeon. This may be possible, as these are specific manual skills that are imprinted in the brain. Access to the hippocampus and cerebellum for memory injection would also be required, as well as the cerebellum and basal ganglia for complex motor tasks.

This would require highly accurate data transmission, which would in some ways be similar to today’s extremely precise computer data transmission, accompanied by instantaneous thought-activated Internet access, or B/CI. The first proof-of-principle of “instant learning” was accomplished using decoded fMRI, where human visual cortex brain activity patterns were induced to match a previously known target state and improve the performance of visual tasks (Shibata et al., 2011). Transcranial magnetic stimulation, involving the application of a strong pulsed magnetic field from outside the skull using a magnetic coil precisely positioned over the head, was also employed to induce new skills. Stimulating a “virtual lesion” of small regions of the brain either diminished or enhanced skills in some transcranial magnetic stimulation experiments, with approximately 40% of participants displaying remarkable new skills, such as drawing abilities (Mottaghy et al., 1999).

### Enhancement of Human Intelligence

The brains of humans with high IQ are extensively integrated with neural pathways that connect distant brain regions, while the brains of humans with low IQ have less-integrated connectivity with shorter neural routes (Colom et al., 2007; Haier and Jung, 2007). Neuralnanorobotically mediated B/CI systems may enable significantly increased human intelligence, eventually superseding the inherent architectures of the brain’s neural domains. Such systems could expand memory capabilities considerably, improve pattern recognition and cognition through the creation of novel hybrid biological/non-biological networks, and interface with non-biological networks as well as new forms of AI.

Neural prostheses are currently employed in cochlear implants to treat hearing loss, as stimulating electrodes to treat Parkinson’s disease and other neurological diseases, and in “artificial retinas” to restore vision, among other applications (Dobelle, 2000; Mayberg et al., 2005; Perlmutter and Mink, 2006; Gaylor et al., 2013; Lewis et al., 2015, 2016). Brain implants employed in locked-in patients permit extraction of brain data into an external computer, enabling patients to communicate with the outside world (Hochberg et al., 2012). Since the hippocampus plays a critical role in learning and memory, damage to this small organ can disrupt proper electrical signaling between nerve cells, impeding the formation and recall of memories. This is something that artificial-brain-inspired prosthetics are currently beginning to treat (Berger et al., 2005, 2011; Lebedev and Nicolelis, 2006).

Computerized implants receiving signals from thousands of brain nerve cells may wirelessly transmit the data to an interfacial device that decodes intentions, with preliminary versions of these implants being used to control artificial limbs (Ferris, 2005; Au et al., 2007; Gordon and Ferris, 2007; Hargrove et al., 2013; Tabot et al., 2013). Neuralnanorobots may offer significant advantages over current surgically installed neural prosthetics, since they might be introduced through the bloodstream without surgery, via a fully reversible procedure that could be reprogrammed in real-time to permit instantaneous software updates.

### Artificial Intelligence and Existential Risk Prevention

Empowered by the exponential increase in price/performance of computational data storage and processing power, artificial intelligence (AI) algorithms are improving across many domains and demonstrating superior capabilities when compared to those of humans. Examples of the superiority of AI include: game-playing (Jeopardy, Go, chess), driving cars, providing diagnostics for some cancer patients, and other examples in various domains (Ferrucci et al., 2010; Levinson et al., 2011; Chouard, 2016). Over the next decade, narrow artificial intelligence algorithms are expected to outperform humans in many other areas. Advances in artificial intelligence across machine learning, machine vision, and natural language processing domains, combined with advances in big data and robotics, are anticipated to empower robots to outperform humans in many, if not most, physical and cognitive tasks. However, in the future, we can expect far more powerful “artificial general intelligence” (AGI), a subfield of AI oriented toward creating thinking machines with general cognitive capability at the human level and beyond. (Minsky, 1985; Nakashima, 1999; Horst, 2002; Hutter, 2005; Goertzel, 2006; Adams et al., 2011).

Interfacing the human brain with the cloud via neuralnanorobotic technologies may be beneficial for humanity by assisting in the mitigation of the serious existential risks posed by the emergence of artificial general intelligence (Bostrom, 2002, 2013; Whitby and Oliver, 2000; Joy, 2007; Bostrom and Cir, 2008; Yudkowsky, 2008; Schneider, 2009). One such mitigation might involve the merging human brains with computers to prevent the dangers of unbridled artificial general intelligence (Dewey, 2015). Neuralnanorobotics may indeed be a suitable technology to assist with reducing human existential risk potentially initiated by rapidly emerging artificial general intelligence by enabling the creation of an offsetting beneficial human augmentation technology.

### Virtual and Augmented Reality

Fully immersive virtual reality may become indistinguishable from reality with the emergence of neuralnanorobotics, rendering many forms of physical travel obsolete. Office buildings might be replaced by virtual-reality (VR) environments in which conferences could be attended virtually, replacing today’s VoIP conference calls and Internet-based video conference calls with highly realistic, fully immersive VR conferences in virtual-reality spaces. Immersive VR may enable long-distance communications in engaging ways within environments that are indistinguishable from reality. The economic and environmental benefits of significantly reducing travel requirements may be significant. For example, Cisco has reported savings of millions of dollars through the use of highly realistic telepresence systems.

Current systems for fully immersive virtual reality include VR headsets and haptic controllers (typically to facilitate immersive gaming) (Alkhamisi and Monowar, 2013; Tweedie, 2015). In principle, fully immersive VR may benefit from advanced neuralnanorobotics to provide, for example, appropriate “proximal cues.”

Neuralnanorobotically induced artificial signals may be indistinguishable from actual sensory data that is being received from the physical body. All brain output signals might be suppressed by neuralnanorobots to avoid the movement of real limbs, mouth, or eyes during virtual experiences; in place of this, virtual limbs would react appropriately while adapting the surrounding virtual world in the field of vision (similar to current immersive gaming). B/CI users might initially encounter a virtual dashboard in the cloud where they can select from an extensive menu that is replete with experiential pathways. The gaming industry provides virtual environments for humans to explore, from recreations of actual locations to fanciful environments — even environments that violate the laws of physics. Virtual trips in simulations of “real” locations will permit the equivalent of nearly instantaneous time travel. Ultrahigh-resolution, fully immersive VR might also enhance business negotiations and web-dating, among other applications. The “real” and the “virtual” worlds could evolve to become practically impossible to distinguish.

Another application of neuralnanorobotics might be manifest as augmented reality—superimposing information about the real world onto the retina to provide real-time guidance, explanations, or data on social events while traveling. Neuralnanorobotics might provide real-time auditory translation of foreign languages, or access to many forms of online information, which would integrate these augmentations into our daily activities. Some types of information might be presented by virtual assistants or avatars that overlay the real world to assist their human partners with the retrieval of information. These virtual assistants, running on the cloud, similarly to IBM Watson, might not even wait for questions if they can predict human desires based on previously registered behavioral patterns and other data.

### Ultrahigh-Resolution Fully Immersive “Transparent Shadowing”

Neuralnanorobotically empowered B/CI technologies accompanied by supercomputing technologies might permit users to experience fully immersive, real-time episodes of the lives of any willing human participant on the planet, via non-intrusive “Transparent Shadowing (TS).” In TS, an individual might literally experience another person’s life, through their own eyes, for a predetermined duration via an “extra life” session. Such a capacity may be anticipated to elevate human collaboration, understanding, respect, and empathy to previously unimaginable levels (Domschke and Boehm, 2014). “We will be able to change our appearance and effectively become other people” (Kurzweil, 2005).

With neuralnanorobotically enabled B/CI, individuals might engage in the TS of voluntary or remunerated “spatial hosts.” Under strict protocols, accredited spatial hosts would agree to allow single or multiple attendees (conceivably numbering in the millions) to literally experience portions of their life experiences over a predetermined timeline/schedule. These TS sessions might be akin to today’s seminars or lecture series, where the knowledge or specific skills of the host would be experientially imparted to the “attendees.” However, these TS sessions would offer exponentially higher resolution in every respect. The full sensorial realm (e.g., physical presence, tactile sensations, olfactory, visual, tastes, and auditory) would be experienced by the attendees, as if they inhabited the body of the spatial host. Although they would perceive the vocal instructions of the host, to temporally experience exactly what the spatial host is experiencing, for the sake of personal privacy, attendees might, by default, be completely blocked from any access to the thoughts, emotions, or self-speak of their spatial hosts (Ford, 2010).

From another perspective, access to some level of self-speak may be beneficial for attendees toward conveying the thought processes/intentions of a spatial host that underly their activities. However, it is likely that any self-speak of a spatial host will include their most private and intimate thoughts. Hence, this warrants a careful exploration of how these self-speak items might be screened such that the attendees are not privy to them, how will this be decided, and by whom. What self-speak will be allowable and what will be considered as out-of-bounds? For this assessment to take place via AI, the self-speak in question would have to somehow be processed in real time/on the fly, as any records of such in any form, would most likely be considered as highly unethical. This may translate to the establishment of a very brief (∼millisecond) latency, from spatial host to attendee, to allow for this virtually instantaneous self-speak screening.

Although the attendees would retain their own identities and experience real-time live-feed full immersion into a portion of the host’s life experiences, these guests would have no capacity to control any aspect of the host. This particular B/CI application would be akin to an exponentially enhanced version of attending and viewing a movie, albeit one that is totally controlled by the spatial host. The host will have no mental or physical perception that they are being “shadowed” by the attendees. Hence, in essence, anyone on the planet (who is B/CI enabled) might be engaged as either a spatial host or an attendee.

Once established and potentially utilized by a growing demographic, this capability might have strong potential for conveying profound beneficial implications for human advancement across multiple domains, possibly assisting with the further development of human collaboration and empathy, perhaps eventually leading to the minimization or elimination of most armed conflict.

Given the prospect of virtual, fully immersive TS, issues pertaining to the possibility of immediate or residual (post-TS session) physiological and/or psychological transference arising from the interactions between a spatial host and any given attendee will require careful consideration. In addition to standardized TS operational procedures and safeguards, a range of prudent failsafe protocols should be explored and established for the protection of both the spatial hosts and the attendees. Potential physiological transference issues may arise when two or more individuals are engaged in shared TS activities. For example, in cases of unpleasant pain, the attendee might activate an instantaneous default auto-disengagement protocol once a particular physiological threshold is perceived by the B/CI system.

TS may hold potential to facilitate understanding of the experiences of other people and to significantly increase empathy. Experiencing episodes of the lives of those in other cultures and ethnic groups could promote cross-cultural understanding and tolerance, improving prospects for the reduction of hatred and racism. For example, perhaps those of majority ethnic groups might be more sensitized with the issue of racism against those of minority ethnic groups, once they “experience” it for themselves through TS sessions. Similarly, minorities who experience a majority host might come to realize that many actions perceived by them as purposeful racism were entirely unintentional. Cross-gender experiences might impact real-life relationships between genders, due to increased empathy and understanding. It might be possible that an eventual shift in gender attitudes could lead to decreased gender-related and domestic violence.

Although beyond the scope and space constraints of this paper, we acknowledge that there will likely be several “display modes” available to B/CI users once the technology matures. These may include optional text, imagery, and streaming video displays that are superimposed at customizable locations within the user’s field of vision. It may be likely that via TS, all knowledge-based queries and responses, as well as fully immersive experiences within avatars and other users, could include an optional toggle mode. When this mode is engaged, internalized visualizations might be projected as on to a “float screen” that can be superimposed within the user’s field of vision, where the float screen can be made more prominent in the user’s experience via dynamic fading.

## Conclusion

Human knowledge is being digitized at an accelerated exponential pace for storage and processing in the cloud. Given our biologically constrained cognitive abilities, the impossibility of the human mind to keep pace with the increasingly rapid generation of human knowledge is evident. Hence, it is essential, and may indeed become urgent, that we develop a safe, robust, stable, secure, and continuous real-time interface system between the human brain and the data storage and processing systems that reside in the cloud. Neuralnanorobotics may provide a technology at the appropriate scale, with a suitable level of complexity to robustly interface the human brain with the massive volume of data that is stored and processed in the cloud.

Neuralnanorobotics strategies involve the direct, comprehensive monitoring of the ∼86 × 10^9^ neurons of the human brain, as well as its ∼2 × 10^14^ synapses and ∼84 × 10^9^ glial cells. Three proposed classes of neuralnanorobots (endoneurobots, gliabots, and synaptobots) may employ ∼3375 nm^3^ FET-based neuroelectric nanosensors to detect and monitor virtually all individual action potentials and their waveforms. Neuralnanorobotic entities would transmit the nominal ∼5 × 10^16^ bits/sec of synaptically processed electronic information, encoded in ∼4 × 10^15^ spikes/sec flowing within the entire living human brain, wirelessly via a nanorobotic auxiliary 30 cm^3^ volume nanoscale fiber-optic system that is capable of handling ∼10^18^ bits/sec. This may permit real-time brain-state monitoring and data extraction into an external supercomputer that communicates directly with the cloud.

A human B/CI system mediated by neuralnanorobotics could empower individuals with instantaneous access to all cumulative human knowledge available in the cloud and significantly improve human learning capacities and intelligence. Further, it might transition totally immersive virtual and augmented realities to unprecedented levels, allowing for more meaningful experiences and fuller/richer expression for, and between, users. These enhancements may assist humanity to adapt emergent artificial intelligence systems as human-augmentation technologies, facilitating the mitigation of new challenges to the human species. Human B/CI systems mediated by neuralnanorobots might also upgrade mutual human understanding and collaboration by making it possible to engage humans in TS experiences, which could enable considerably improved understanding and tolerance among all members of our diverse and amazing human family.

## Author Contributions

All authors listed have made a substantial, direct and intellectual contribution to the work, and approved it for publication.

## Conflict of Interest Statement

YS of NanobotMedical, Inc. declares no competing or conflicting interests and FB of NanoApps Medical, Inc. declares no competing or conflicting interests. The remaining authors declare that the research was conducted in the absence of any commercial or financial relationships that could be construed as a potential conflict of interest.

## Footnotes

1 For the purposes of this paper, the term “invasive” is defined as a medical procedure or device that imparts quantifiable physiological damage (at any level) to a patient. In the case of the envisaged nanomedically enabled B/CI, the assumption is that millions of micron-scale nanorobots will be non-invasive — i.e., they may ingress into a patient and subsequently auto-situate themselves at various sites within the human brain, with no disruptive functional physiological or experiential effects. Or in some cases, they may be minimally invasive.

2 Alternatives to the regular injection of neuralnanorobots into the human vasculature include: intravenously, intranasally in aerosolized form, orally as a pill, via a dermal patch, or topical gel.

## Abbreviations

AIS: Axon initial segment
B/CI: brain/cloud interface
BCI: brain–computer interface
BMI: brain–machine interface
BTBI: brain-to-brain interface
EEG: electroencephalography
fMRI: functional magnetic resonance imaging
FNIRS: functional near-infrared spectroscopy
TS: transparent shadowing
